# Unmanned Aerial Vehicle to Estimate Nitrogen Status of Turfgrasses

**DOI:** 10.1371/journal.pone.0158268

**Published:** 2016-06-24

**Authors:** Lisa Caturegli, Matteo Corniglia, Monica Gaetani, Nicola Grossi, Simone Magni, Mauro Migliazzi, Luciana Angelini, Marco Mazzoncini, Nicola Silvestri, Marco Fontanelli, Michele Raffaelli, Andrea Peruzzi, Marco Volterrani

**Affiliations:** 1 Department of Agriculture, Food and Environment, University of Pisa, Pisa, Italy; 2 GLOBI Hi-Tech Srl, Genova, Italy; Chongqing University, CHINA

## Abstract

Spectral reflectance data originating from Unmanned Aerial Vehicle (UAV) imagery is a valuable tool to monitor plant nutrition, reduce nitrogen (N) application to real needs, thus producing both economic and environmental benefits. The objectives of the trial were i) to compare the spectral reflectance of 3 turfgrasses acquired via UAV and by a ground-based instrument; ii) to test the sensitivity of the 2 data acquisition sources in detecting induced variation in N levels. N application gradients from 0 to 250 kg ha^-1^ were created on 3 different turfgrass species: *Cynodon dactylon x transvaalensis* (*Cdxt*) ‘Patriot’, *Zoysia matrella* (*Zm*) ‘Zeon’ and *Paspalum vaginatum* (*Pv*) ‘Salam’. Proximity and remote-sensed reflectance measurements were acquired using a GreenSeeker handheld crop sensor and a UAV with onboard a multispectral sensor, to determine Normalized Difference Vegetation Index (NDVI). Proximity-sensed NDVI is highly correlated with data acquired from UAV with r values ranging from 0.83 (*Zm*) to 0.97 (*Cdxt*). Relating NDVI-UAV with clippings N, the highest r is for *Cdxt* (0.95). The most reactive species to N fertilization is *Cdxt* with a clippings N% ranging from 1.2% to 4.1%. UAV imagery can adequately assess the N status of turfgrasses and its spatial variability within a species, so for large areas, such as golf courses, sod farms or race courses, UAV acquired data can optimize turf management. For relatively small green areas, a hand-held crop sensor can be a less expensive and more practical option.

## Introduction

Regarding turfgrass management a concepts, that is related to the variation in soil characteristics and climate, plant adaptability and irrigation, is Precision Agriculture (PA) [1; 2]. PA is the application of geospatial techniques and sensors such as Global Positioning System (GPS), Geographic Information System (GIS), and remote sensing, i) to identify variations within the field and to deal with them using alternative strategies; ii) to apply inputs (e.g. water, fertilizer, and pesticides) strictly where, when, and in the amounts needed by plants [3; 4]. Another important concept, related to PA is Precision Turfgrass Management (PTM) useful to monitor pests, fertilization, salinity stresses and irrigation problems [5; 6; 7]. For crop agriculture, optical sensing and in particular spectral reflectance have become important parts of PTM [8], as the analysis of radiation reflected by plants (including turfgrasses) can supply information on species quality [9; 10; 11], leaf area index [12], chlorophyll [13], biomass [14; 15], drought stress [16; 17], and general nutritional status [2; 18; 19; 20; 21]. Nitrogen fertilization on turfgrasses is one of the factors that most influence physiological and aesthetic aspects [11; 18; 22; 23; 24]. Nitrogen represents also an important nutrient for maintaining a good quality of turfgrass (green color, density, recovery from drought diseases, and wear stress) [25; 26].

In previous research [27; 16], vegetation indices were calculated by combining various reflectance bands of the spectrum and correlated with relevant turfgrass canopy parameters. In these studies the normalized difference vegetation index (NDVI = (RNIR − Rred)/(RNIR + Rred); RNIR = reflectance in the near infrared region, Rred = reflectance in the red region) was considered as the most commonly used reflectance-based plant stress indicator [28; 29; 30; 31; 32]. NDVI relies on the concept of a relationship between the absorption of visible light and resilient reflectance of near-infrared light to the chlorophyll in vegetation [33; 34, 35, 2]. The range of NDVI varies from 0.00 to 0.99 and correlates positively with turfgrass quality. This index is also influenced by differences in species, environmental stresses, fertilization and pest injuries [36; 37; 2; 9].

High-resolution satellite imagery is commonly used to study the variations for crop, forest and soil conditions [38; 39]. However, the availability and the often prohibitive costs of such imagery, justify an alternative data acquisition for application in PA. Specifically, images taken by low altitude remote sensing platforms, or small unmanned aerial systems (UAS), can be a valid alternative in agriculture, [40] given the low cost of operation, high spatial and temporal resolution, and high flexibility in terms of flight planning and acquisition scheduling [41]. Moreover, UAS are less affected by weather conditions as they can operate also on overcast days and the information can be immediately accessible as a tool for remote sensing scientists and farmers [42]. In recent years, small commercial UAS (<50 kg) [43] have been available for environmental and agricultural applications [44]. Several recent studies report on the application of UAS imagery for PA [45]. Thus, aerial spectrometry from UAV bridges the gap between ground-based observations and remotely sensed imagery from conventional aircraft and satellite platforms [46; 47; 48; 49]. Remote sensors placed on unmanned aerial vehicles (UAVs) could provide low-cost approaches to meet the critical requirements of spatial, spectral, and temporal resolutions [50]. In the past the limited payload has been one major limitation for UAV sensing, resulting in most of applications relying on standard or modified commercial cameras [51; 52]. A multispectral sensor is, however, necessary for estimating biophysical parameters, mapping different vegetation species and identifying vegetation stress conditions [53]. At present, the most largely used sensors are video and multi-band digital cameras, while very few studies have tested the use of hyper-spectral sensors mounted on UAVs [54]. In agriculture UAVs have been mainly employed for image surveys such as mapping invasive weed outbreaks in coffee plantations, finding irregularities in the fertilization delivery system and determining ripeness analysis [55]. UAVs have also been employed in vineyard experiments with the purpose of analyzing soil variability, pest problems, differences in fruit ripeness [56], and monitoring night time temperatures for frost mitigation [57; 58]. Other authors have presented the development of a remote-sensing aerial platform for traffic monitoring [59], rescue mission [60], environment perception [61], and general mapping purposes [62]. 3D-GIS map generation employing UAVs have also been discussed in [63]. Furthermore remote sensing for real-time water management and irrigation control with small UAV is proposed in [64]. To date, spectral reflectance acquisition for turfgrass research or management has been carried out mostly by aerial or proximity devices. Taking a broader view, the potential large-scale management and control of turfgrass fertilization using remotely sensed UAV data is desirable [2].

The objective of the trial was (a) to compare the spectral reflectance through the acquisition of NDVI on three different turfgrass species via Unmanned Aerial Vehicle with onboard a digital camera and by a ground-based instrument; (b) to test the sensitivity of the two data acquisition sources in detecting induced variation in nitrogen levels on different turfgrass surfaces.

## Materials and Methods

The trial was carried out from June to September 2015 in S. Piero a Grado, Pisa, at the Department of Agriculture, Food and Environment of the University of Pisa (43°40’N, 10° 19’E, 6 m. a.s.l.) on mature turfgrass stands of *Cynodon dactylon x transvaalensis (Cdxt)* ‘Patriot’, *Paspalum vaginatum (Pv)* ‘Salam’ and *Zoysia matrella (Zm)* ‘Zeon’.

The swards were all established on a calcaric fluvisoil (Coarse-silty, mixed, thermic, Typic Xerofluvents) with pH 7.8 and 18 g kg^-1^ of organic matter.

As described in detail previously by [2] in year 2015, no fertilizer had been applied to the turfgrass before the trial started. In order to create a linear nitrogen gradient, on June 16, 2015 fertilization was carried out applying ammonium sulphate with a Scotts AccuPro 2000 rotary spreader, obtaining for each turfgrass 14 different application rates ranging from 0 to 250 kg ha^-1^ of N (increases of 10 kg ha^-1^ of N every 1 m). Very high N rates have been applied in order to reach the saturation of NDVI values, regardless of the practical benefits to turfgrasses. For each species the experimental area was: 30 m x 4 m x 4 replications (480 m^2^). After the fertilization, 5 mm of water was applied to incorporate the fertilizer into the soil. During the trial period a turf height of 2.0 cm was maintained by mowing with a walk-behind reel mower (John Deere 20SR7, Moline IL, USA) and the clippings were removed. In the entire experimental area, in order to evaluate nitrogen fertilization as the only variability source, identical irrigation and maintenance practices were applied. During the trial no weed or pest control was necessary [2].

On July 6, 2015, within the linear gradient 14 equally spaced (2 m) sampling zones were identified and on each species 56 (14 x 4 replications) proximity and remote sensed readings were acquired starting from the unfertilized control to the highest nitrogen rate, with an Unmanned Aerial Vehicle with a digital camera onboard and a ground-based handheld crop sensor. Data acquisition was carried out in the above mentioned day because the effect of nitrogen fertilization on turfgrass had to be evident and measurable by the two instruments.

Spectral measurements were taken between 11.30 am and 1.30 pm (local time), in complete absence of clouds. The weather parameters were as follows: avg. air temperature 27.8°C, avg. relative humidity 75%; Photosynthetic Photon Flux Density 1540 μmol/m^2^/sec; avg. wind speed 4.2 km/h. Each ground-based measurement was geo-referenced to sub-metre accuracy with a Differential Global Positioning System receiver, in order to retrieve the exact location on the UAV imagery [2].

### UAV flight and image processing

**Fig 1:**
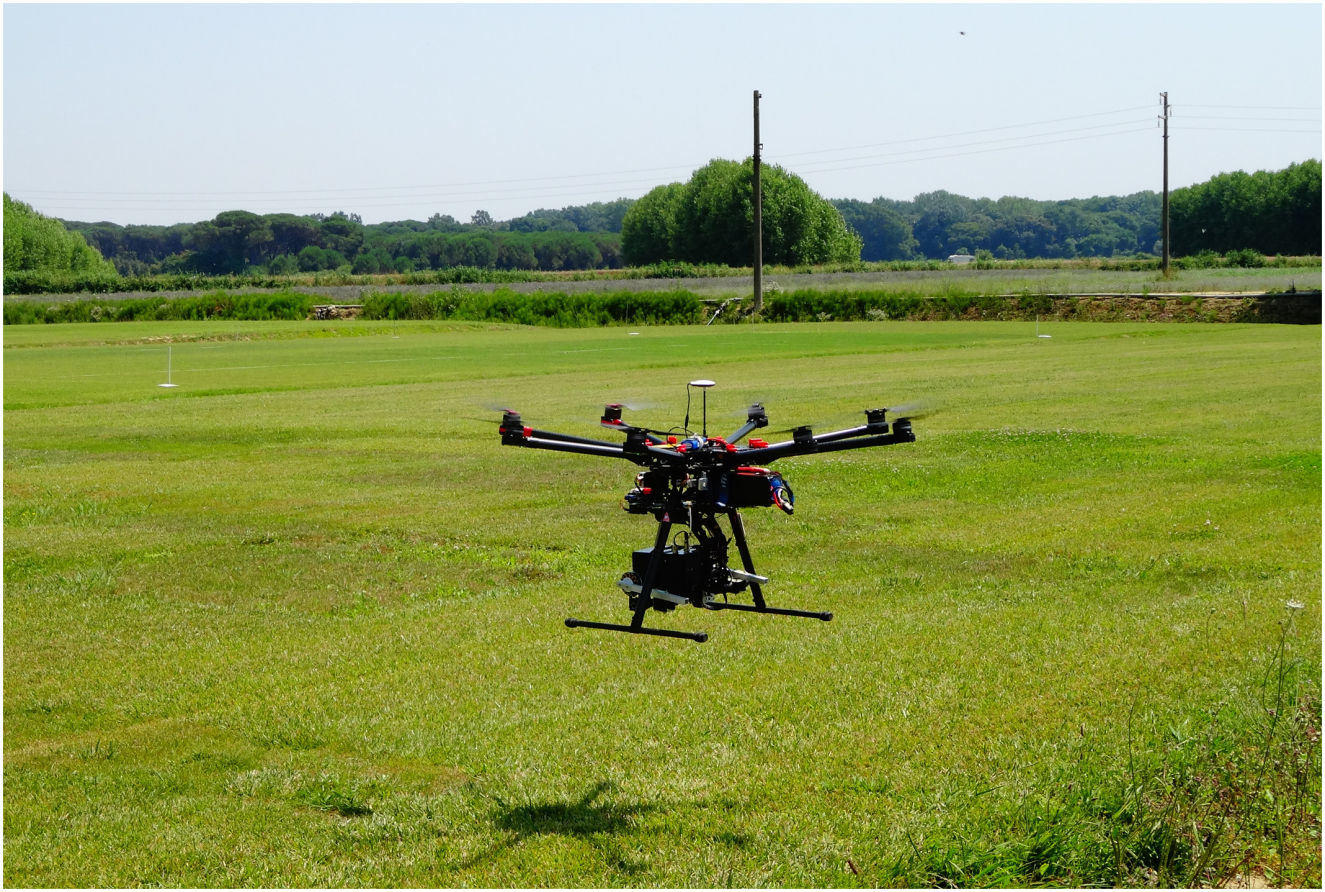
The UAV during flight operations (July 6, 2015; Pisa, Italy; 43°40’N, 10° 19’E, 6 m. a.s.l.) and a detail of the vehicle DJI S1000 Octocopter.

On July 6, 2015 a DJI S1000 Octocopter (DJI, Shenzhen, China) (Fig 1) equipped with a digital commercial camera (Canon S100, Canon Inc., Tokyo, Japan) and a lightweight multispectral sensor (Tetracam ADCMicro, Tetracam, Inc., Chatsworth, CA, USA). It has flown above the experimental area at 50 m above ground and both visible photographs and a multispectral image have been acquired with a total of 200 frames to cover the turfgrass fields. The spatial resolution of the output data was about 5 cm.

Geometric calibration of the sensor was performed using Automatic Photogrammetric Processing Station (Menci Software), a very rigorous environment to test the real potentiality of new integrated VectorNav board VN-200 GPS / IMU built-in multispectral sensor [65].

**Fig 2:**
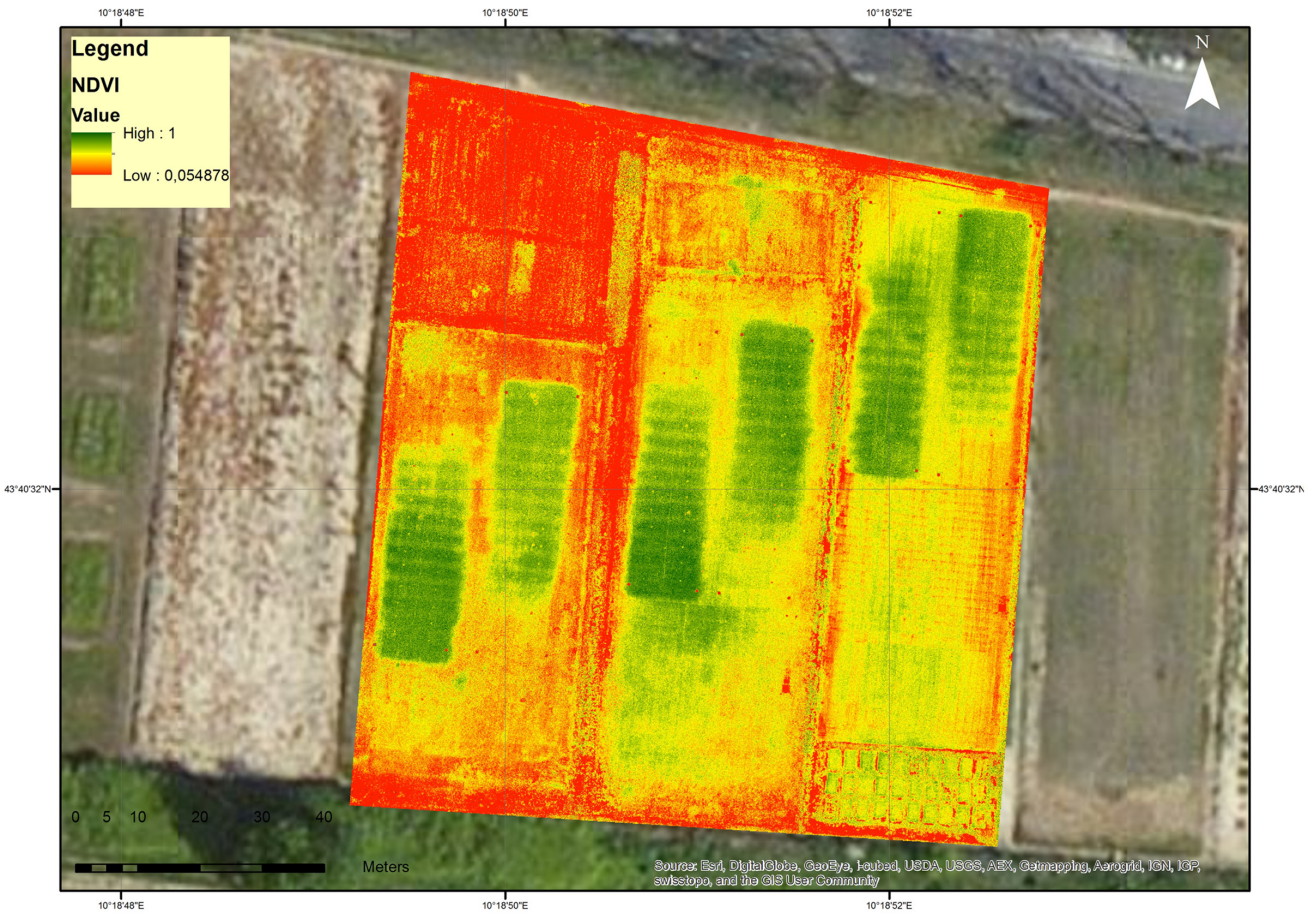
The RGB image of the turfgrass fields acquired by Tetracam ADCMicro mounted on the unmanned aerial vehicle.

A radiometric calibration has been also performed with a three steps normalization procedure: i) estimation of across-track illumination variability by using smoothed (20 m kernel) band-averaged response, ii) normalization of band-averaged response upon mean value of across-track Regions Of Interest, and iii) inversion of band-averaged rescaling to produce a normalization layer. The derived normalization layer was multiplied by the original digital number to derived normalized data in the green, red and NIR (Near InfraRed) channels. Normalized digital number channels were then calibrated to surface reflectance values using empirical line regression and in situ spectral measurements. Reflectance is measured in 3 multi-spectral bands: Green 520–600 nm; Red 630–690 nm; Near Infrared (NIR) 760–900 nm. The Normalized Difference Vegetation Index (NDVI) was derived from the normalized and calibrated reflectance of the three sensor’s channels (Green channel, Red channel, NIR channel). Every pixel (0.05 x 0.05 m) of the image contained coordinates and an NDVI value (Fig 2). Pixel NDVI values were extracted using ENVI software (RSI Inc., Boulder, CO, USA). Thus, the plots were identified and NDVI values were obtained in the same position where the ground NDVI readings were performed [2].

### Field data

On the same day of UAV flight, ground-based measurements of spectral reflectance were carried out with a handheld crop sensor at a height of 110 cm from the ground, thus monitoring a surface of about 2000 cm^2^ (Ø = 50cm). The Trimble’s GreenSeeker Handheld Crop Sensor, Model HSC-100 (Trimble Navigation Unlimited, Sunnyvale, CA) has an active light source that makes readings unaffected by sunlight [66]. Reflectance is measured in the red region at 660 nm, ~25 nm Full Width at Half Maximum and in the near infrared region of the spectrum at 780 nm ~25 nm Full Width at Half Maximum. The output of GreenSeeker is directly provided as NDVI value.

Immediately after the remote and proximity sensed measurements and in the same area where the optical readings were carried out, the following parameters were observed:

- surface temperature (°C): an infrared thermometer (Testo mod. 825-T2) was placed 0.8 m above the surface in order to collect the surface temperature;
- color intensity (from 1 = very light green to 9 = very dark green): visual assessments [67].

Furthermore samples of clippings were collected on each sampling zone with a walk-behind reel mower from a surface of 0.5 m^2^ (1 x 0.5 m). Fresh clippings were put in a ventilated stove at 70°C, dried to constant weight, and the total nitrogen (N) was determined by the micro-Kjedahl method [68].

### Statistical analysis

The relationship among the two different NDVI reading methods (ground-based with GreenSeeker and remoted with UAV), surface temperature, color, applied nitrogen and clippings nitrogen content were studied using CoStat software (CoHort, Monterey, CA, USA) and Pearson’s correlation coefficients (r) were calculated. In particular, the correlations between the two different reading methods of NDVI, N applied to turf, and clippings N content were studied in order to verify whether (a) UAV imagery could be useful as a diagnostic tool to identify variation in N status of turfgrass; and (b) NDVI UAV data are suitably correlated with data obtained from ground-based sensor.

Linear regression equations were studied for the correlations showing significant coefficients.

## Results

### Cynodon dactylon × transvaalensis (Cd×t)

For correlations (r) among NDVI values obtained with the two different instruments and the N rates applied to the *Cd×t* plots, the highest value was found for N/NDVI (UAV) (r = 0.91), though N/NDVI(GreenSeeker) (r = 0.86) was still highly correlated with the levels of N applied (Table 1). Comparing NDVI values obtained with the GreenSeeker and with the UAV, the r value is significantly correlated (r = 0.97) (Table 1).

**Table 1:** Pearson product-moment correlation coefficients (r) among the nitrogen applied, clippings nitrogen content, color intensity, temperature, NDVI measured with a ground-based instrument (Trimble’s GreenSeeker sensor) and NDVI measured with an unmanned aerial vehicle (UAV) on a) *Cynodon dactylon x transvaalensis*; b) *Paspalum vaginatum*; c) *Zoysia matrella*. In each species correlation coefficients are calculated across all entries.

*r*	N applied	N (%) clippings	Color intensity (1–9)	Temperature (°C)	NDVI GreenSeeker (780,660)	NDVI UAV (830,660)
*a) Cd x t*						
N applied (kg ha^-1^)	-	0.94^★★★^	0.91^★★★^	NS	0.86^★★★^	0.91^★★★^
N (%) clippings	-	-	0.93^★★★^	- 0.33^★^	0.91^★★★^	0.93^★★★^
Color intensity (1–9)	-	-	-	- 0.29^★^	0.95^★★★^	0.96^★★★^
Temperature (°C)	-	-	-	-	- 0.32^★^	- 0.34^★^
NDVI GreenSeeker (780,660)	-	-	-	-	-	0.97^★★★^
NDVI UAV (830,660)	-	-	-	-	-	-
*b) Zm*						
N applied (kg ha^-1^)	-	0.94^★★★^	0.95^★★★^	NS	0.82^★★★^	0.79^★★★^
N (%) clippings	-	-	0.95^★★★^	- 0.37^★★^	0.85^★★★^	0.81^★★★^
Color intensity (1–9)	-	-	-	- 0.26^★^	0.86^★★★^	0.84^★★★^
Temperature (°C)	-	-	-	-	- 0.34^★^	NS
NDVI GreenSeeker (780,660)	-	-	-	-	-	0.83^★★★^
NDVI UAV (830,660)	-	-	-	-	-	-
*c) Pv*						
N applied (kg ha^-1^)	-	0.93^★★★^	0.91^★★★^	NS	0.86^★★★^	0.79^★★★^
N (%) clippings	-	-	0.92^★★★^	NS	0.91^★★★^	0.87^★★★^
Color intensity (1–9)	-	-	-	- 0.41^★★^	0.92^★★★^	0.90^★★★^
Temperature (°C)	-	-	-	-	- 0.28^★^	- 0.28^★^
NDVI GreenSeeker (780,660)	-	-	-	-	-	0.96^★★★^
NDVI UAV (830,660)	-	-	-	-	-	-

^★^ = Significant at 0.05 level;

^★★^ = Significant at 0.01 level;

^★★★^ = Significant at 0.001 level; NS = not significant.

**Fig 3:**
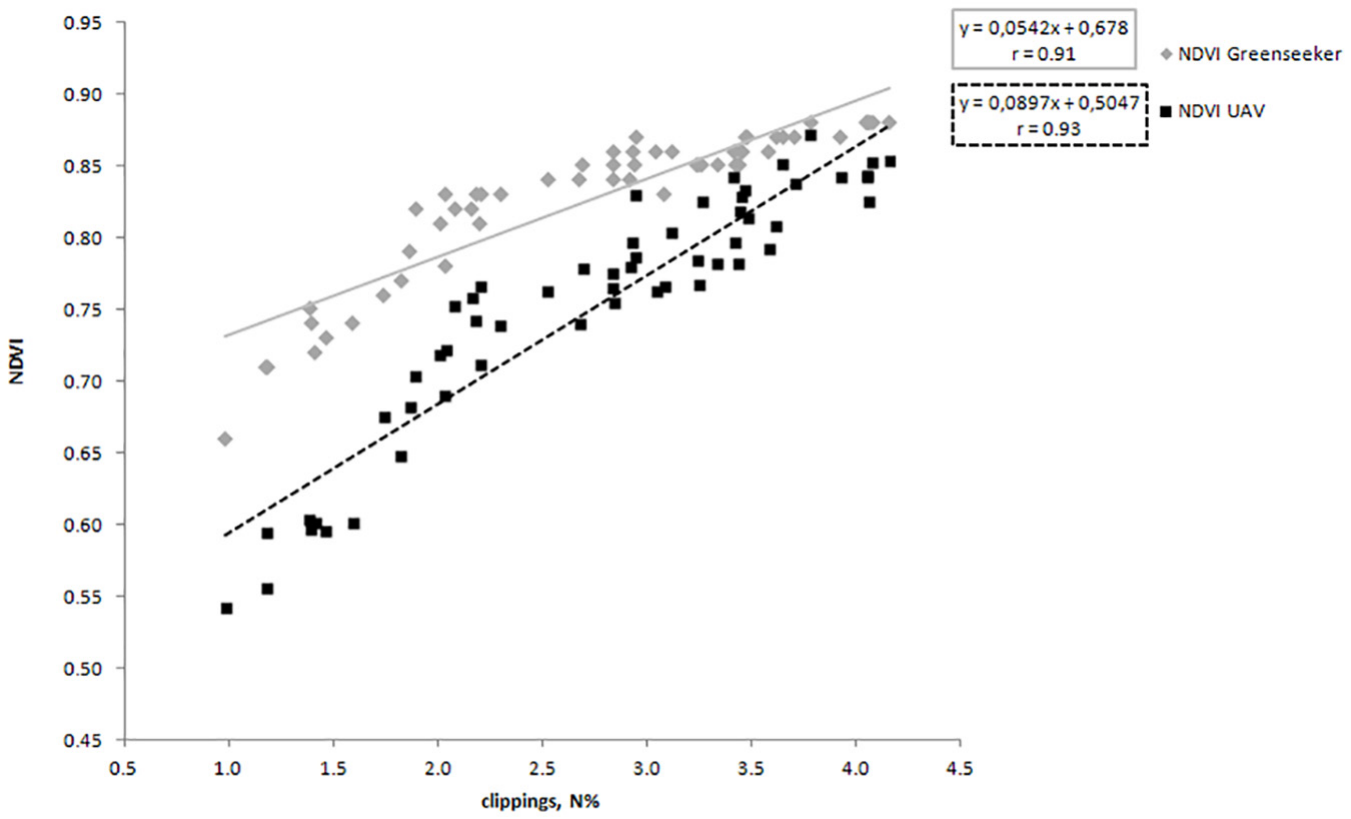
Relationships between the clippings nitrogen content of *Cynodon dactylon x transvaalensis* and NDVI data obtained with a ground-based spectral reflectance instrument (Trimble’s GreenSeeker sensor) and an unmanned aerial vehicle (UAV). Values represented the 4 replications.

Furthermore, observing the correlations, the parameter color intensity is well correlated with both NDVI GreenSeeker (r = 0.95) and NDVI from UAV (r = 0.96). Moreover, as expected, the relationship between color intensity and clippings N content is also significantly high (r = 0.93). The surface temperature is found to be not significant in the correlation with N applied and significant at 0.05 level with clippings N content, color intensity and with NDVI measured with the two spectral sensors. Fig 3 shows the regression line between the clippings N content and both NDVI (GreenSeeker) and NDVI (UAV). Values observed in the figure are obtained from all the 4 replications. It is of interest to note that the regression coefficients are high for both the instruments. However, NDVI (UAV) showed the highest degree of association with clippings N content (r = 0.93).

### Zoysia matrella (Zm)

In the statistical comparisons between the N applied to *Zoysia matrella* (*Zm*) plots and NDVI obtained with the two different instruments, the highest coefficient (r = 0.82) was found for NDVI (GreenSeeker) (Table 1), though N/NDVI (UAV) (r = 0.79) was still well correlated with the levels of N applied on the surfaces.

In regard to the correlation between NDVI values obtained with the two different instruments, the r value was found to be significant (r = 0.83) (Table 1).

**Fig 4:**
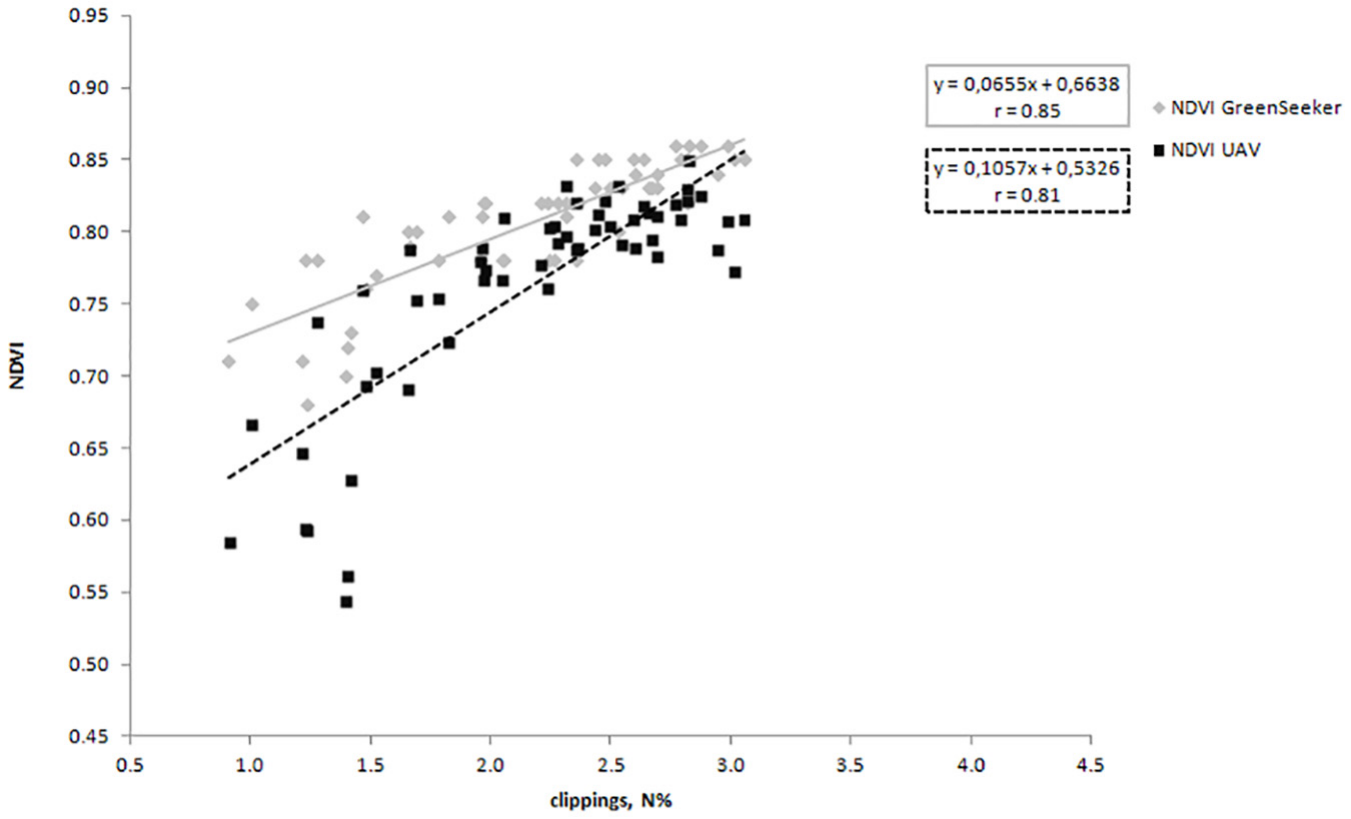
Relationships between the clippings nitrogen content of *Zoysia matrella* and NDVI data obtained with a ground-based spectral reflectance instrument (Trimble’s GreenSeeker sensor) and an unmanned aerial vehicle (UAV). Values represented the 4 replications.

Among Pearson’s correlation coefficients observed, color intensity is, as expected, highly correlated with N applied on the surface and with clippings N content (r = 0.95). Furthermore color is well correlated also with the NDVI obtained with the two radiometric instruments (r > 0.80). Also in this species the surface temperature measures with an infrared thermometer is found to be low significant in the relationship with N on the clippings, color intensity and NDVI measured with GreenSeeker. Relating the surface temperature with the nitrogen applied on the surface and with NDVI obtained with UAV the correlation coefficients are not significant (Table 1). Among the relationships observed by relating NDVI values obtained with the two instruments and the clippings N content, the highest value was still found for NDVI (GreenSeeker) (0.85) (Fig 4), though even in this case also NDVI obtained with the UAV is well correlated with the nitrogen content present in the clippings (0.81).

### Paspalum vaginatum (Pv)

The study of statistical comparisons performed on *Pv* has shown that NDVI (GreenSeeker) is highly correlated with the N rates applied to the turfgrass (r = 0.86) (Table 1). Relating the NDVI values of the two sensors, the correlation value was found to be high (r = 0.96). Furthermore, the parameter color intensity is well correlated with both NDVI GreenSeeker (r = 0.92) and NDVI from UAV (r = 0.90). Moreover, as expected, the relationship between color intensity and clippings N content is also significantly high (r = 0.92). The surface temperature is found to be not significant in the correlation with N applied and with the clippings N content. It resulted significant at 0.05 level with NDVI measured with the two spectral sensors and at a level of 0.01 with color intensity.

**Fig 5:**
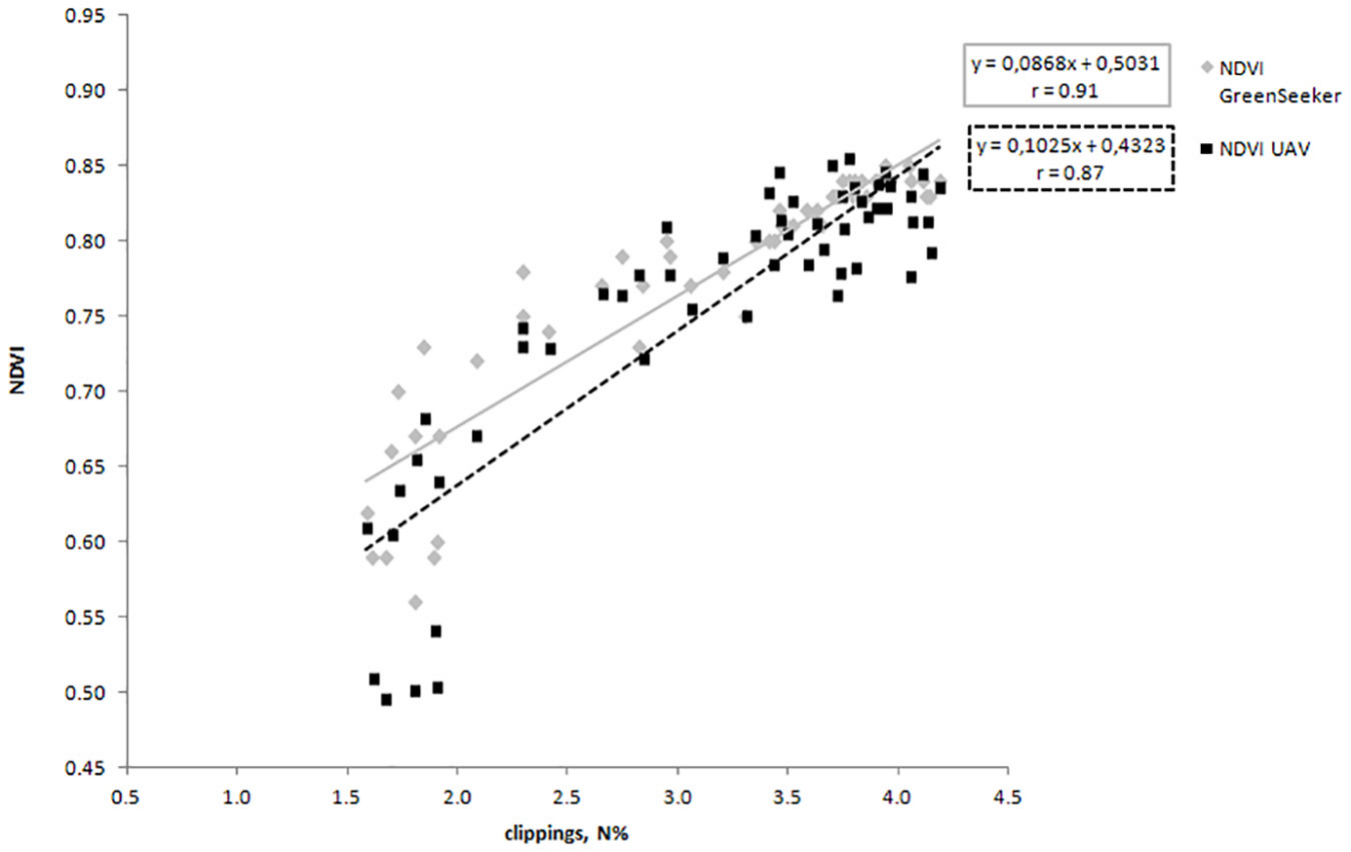
Relationships between the clippings nitrogen content of *Paspalum vaginatum* and NDVI data obtained with a ground-based spectral reflectance instrument (Trimble’s GreenSeeker sensor) and an unmanned aerial vehicle (UAV). Values represented the 4 replications.

In the relationship between the NDVI values obtained with the different instruments and the clippings N content, Pearson’s correlation coefficient was higher with the GreenSeeker (r = 0.91) than with UAV (r = 0.87) (Fig 5).

## Discussion and Conclusions

New technology can bring a unique perspective to turf management. Unmanned aerial vehicles (UAV), or “drones,” can provide valuable information to aid sport turf managers. As part of a management program, the application of drones can save time, labor, and money, helping to highlight, with the use of specific vegetation indices, spectral differences including turf quality, color, dry matter, chlorophyll, carotenoids, N content, and other important information [13; 69; 11].

**Fig 6:**
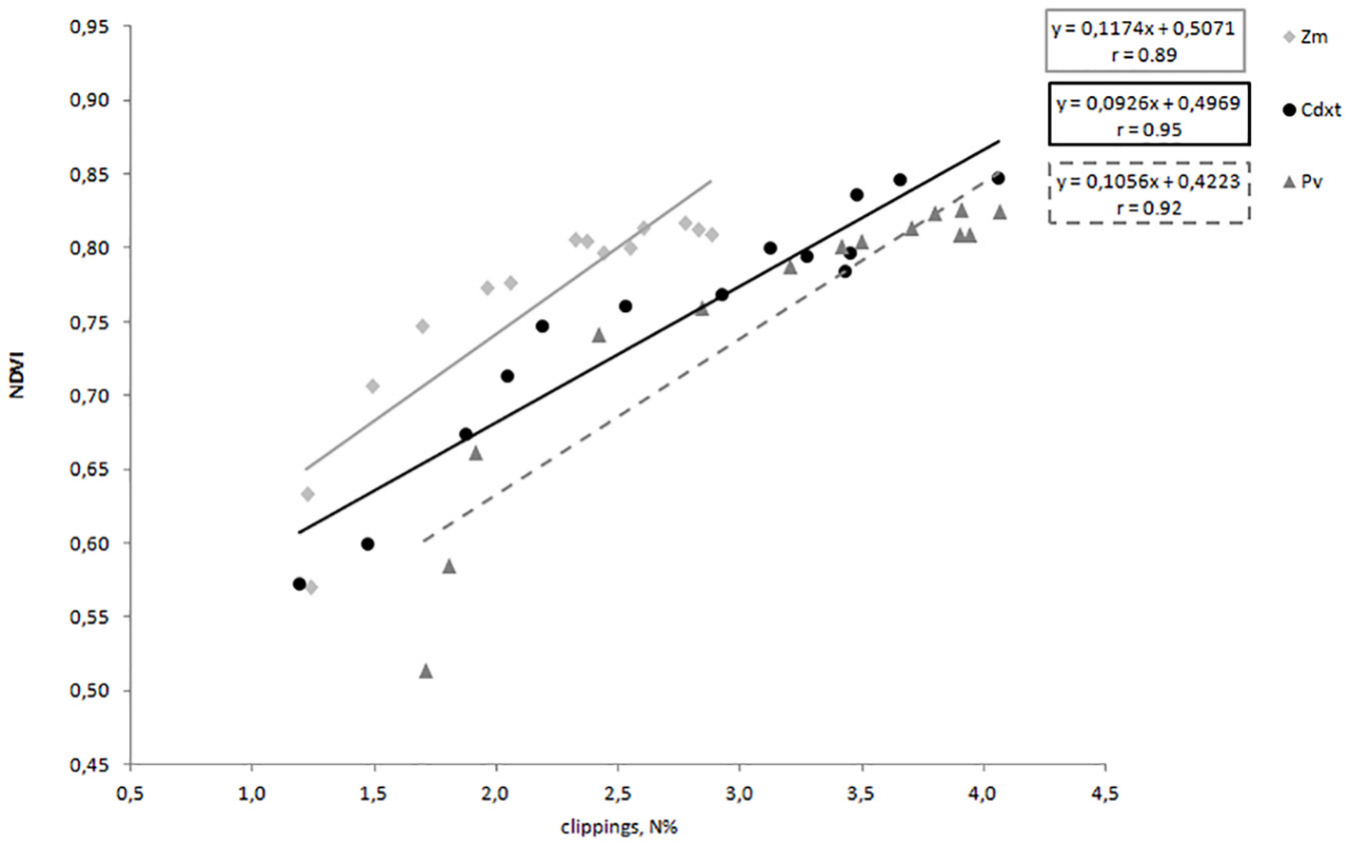
Relationships between the clippings nitrogen content and NDVI data measured with the UAV on *Cynodon dactylon x transvaalensis*, *Paspalum vaginatum* and *Zoysia matrella*. In each species values are means of 4 replications.

In a turf management program, UAV are best used as a platform for collecting aerial imagery. Digital cameras collect visible light reflected from surfaces. Imagery can provide real-time information on many aspects of turf quality important to turf managers. Images can be analyzed with computer software and used to quantify turf status through a process called Digital Image Analysis. This study performed (a) the evaluation of different N rates applied to three turfgrass species and the clippings N content; (b) the comparison of these values with NDVI obtained with two different instruments, one ground-based and one remote. The evaluation of these results showed that NDVI values obtained with the ground-based instrument is highly correlated with UAV spectral data. As found by [28], NDVI was correlated with crop nutrient deficiency, small grain yield, and water stress. However, NDVI has to be considered as a measurement of the effect of many plant growth factors, even in turfgrasses [29; 13; 37]. Evaluating all the correlation data obtained, we notice that the relationships between the clippings N content and NDVI values obtained with the camera onboard the UAV are well correlated. In the relationship between NDVI-UAV and clippings N, the highest r is for *Cdxt* (0.95) (Fig 6), that is also the most reactive species to N fertilization with a clippings N% ranging from 1.2% to 4.1%. Without fertilization, the highest N content is recorded for *Pv* (1.7% N), higher than the other two warmseason turf species (*Cdxt* and *Zm* 1.2% N).

In *Zm* with increasing N rates applied to the turf, plant uptake is significantly lower than *Cdxt* and *Pv*, with a peak value of 2.8% N. Moreover, relating clippings N content and NDVI obtained from UAV, at the highest content of N, the NDVI value in *Cdxt* (NDVI 0.85) is higher than the other two warm-season species *Zm* (NDVI 0.81) and *Pv* (NDVI 0.82) (Fig 6).

Results proved also that proximity-sensed NDVI collected with GreenSeeker is highly correlated with data acquired from UAV with correlation coefficients (r) ranging from 0.83 (*Zm*) to 0.97 (*Cdxt*) (Table 1). UAVs are expected to play an expanded role, complementary to that of satellites and conventionally piloted aircraft in agricultural support [57]. Satellites are useful platforms for regional to global data acquisition [2], yet remain limited in their ability to provide imagery of adequate spatial and temporal resolution for many aspects of commercial agriculture.

UAV imagery can adequately assess the N status of different turfgrasses and its spatial variability within a species. For relatively small areas, such as parks and gardens, a GreenSeeker hand-held crop sensor can be useful in detecting turfgrass stresses because it is a less expensive and more practical option. For larger areas, such as golf courses, sod farms, or turfgrass seed production farms, in addition to the use of proximal sensors it may be necessary to monitor the entire surface using special cameras onboard UAV.

## Abbreviations

GIS: Geographic Information System
GPS: Global Positioning System
NDVI: Normalized Difference Vegetation Index
NIR: Near InfraRed
PA: Precision Agriculture
PC: Precision Conservation
PTM: Precision Turfgrass Management
UAS: Unmanned Aerial Systems
UAV: Unmanned Aerial Vehicle
